# Association between multiple health behaviors and mental health in Chinese college students: a cross-sectional study

**DOI:** 10.3389/fpubh.2026.1792272

**Published:** 2026-05-12

**Authors:** Qingchun Wang, Liu Mu, Guifang Liu, Junming Lyu, Wei Liang, Lin Zhou

**Affiliations:** 1 School of Sport and Health, Shandong Sport University, Jinan, China; 2 School of Graduate Education, Shandong Sport University, Jinan, China; 3 School of Physical Education, Shenzhen University, Shenzhen, China

**Keywords:** college students, health behaviors, mental health, screen time, sedentary behavior

## Abstract

**Background:**

College students face significant psychological pressures, yet the cumulative effects of multiple concurrent health behaviors on their mental health remain underexplored, particularly in the Chinese context.

**Methods:**

A cross-sectional survey was conducted among 521 college students from Shandong and Guangdong provinces, China. Health behaviors, including physical activity (IPAQ-C), sedentary behavior (SBQ), sleep quality (PSQI), smoking, and alcohol use, were assessed via validated self-report questionnaires. Mental health outcomes were measured using the Personal Wellbeing Index (PWI) and the Depression, Anxiety, and Stress Scales-21 (DASS-21). Multiple linear regression analyses, adjusting for key demographic covariates, were employed to examine associations.

**Results:**

Prolonged screen time was consistently associated with poorer mental health, showing negative correlations with personal wellbeing (β = −0.03, *P* = 0.036) and positive correlations with stress (β = 0.24, *P* = 0.030), depression (β = 0.32, *P* = 0.005), and anxiety (β = 0.25, *P* = 0.020). Alcohol consumption was negatively associated with personal wellbeing (β = −0.16, *P* = 0.040). Interestingly, smoking was negatively associated with stress (β = −1.37, *P* = 0.020) and depression (β = −1.44, *P* = 0.016). Physical activity (MVPA) showed no statistically significant associations with stress (β = 0.01, *P* = 0.750), depression (β = 0.01, *P* = 0.630), or anxiety (β = −0.001, *P* = 0.490) in the regression models. Furthermore, no significant gender differences were observed in the key health behaviors or mental health scores.

**Conclusion:**

Unhealthy behaviors, particularly high screen time and alcohol use, are significantly associated with adverse mental health outcomes among Chinese college students. The findings suggest that smoking might be used as a maladaptive coping mechanism. Public health interventions should adopt an integrated approach, simultaneously targeting these co-occurring behavioral risk factors to promote student wellbeing.

## 1 Introduction

In contemporary society, college students represent a critical cohort shaping national development, with their physical and mental wellbeing significantly influencing both individual growth and long–term societal progress. During this transitional life stage, they often face multifaceted challenges, including academic pressure, interpersonal difficulties, and career uncertainty (1). Intensified academic demands, ambiguous career prospects, and ongoing identity exploration further compound psychological and physiological stress (2). Against the backdrop of accelerating globalization and digitalization, the pace of campus life has also escalated, rendering health–related issues increasingly prominent among this population (2).

Various unhealthy behaviors are prevalent among college students today. These habits not only harm their physical health but also profoundly impact their mental wellbeing, often serving as significant triggers for psychological issues (3). Research indicates that college students generally engage in insufficient physical activity (PA), spending much of their free time on electronic devices, which contributes to increasingly sedentary lifestyles (4–6). Additionally, disrupted sleep patterns are common, with behaviors such as staying up late, oversleeping, binge-watching movies, and gaming disturbing biological rhythms and hindering natural physical and mental recovery (7).

Unhealthy behaviors significantly impact college students' mental health (8, 9). Psychological issues including anxiety, depression, and excessive stress have become increasingly prevalent among this demographic severely compromising their academic efficiency, quality of life, and social adaptability. Research indicates that mental health problems not only directly impair academic performance and the development of interpersonal relationships but may also exert long-lasting adverse effects on career development and overall life satisfaction (10).

It is noteworthy that the concept of health behaviors in this study does not represent a simple aggregation of individual actions; rather, it constitutes a multidimensional behavioral system that encompasses various health-related patterns. According to existing research, health behaviors are commonly categorized into two major types: health-promoting behaviors and health-risk behaviors (11). Health-promoting behaviors refer to actions aimed at maintaining and enhancing physical and mental wellbeing through proactive lifestyle choices and preventive measures, such as regular exercise, balanced nutrition, adequate sleep, and active social engagement. Existing evidence indicates that these behaviors alleviate symptoms of anxiety and depression while improving overall mental health (12). In contrast, health-risk behaviors include smoking, alcohol consumption, and prolonged sedentary behavior (SB), which increase the likelihood of disease or other health issues. Multiple studies have demonstrated a significant positive correlation between such behaviors and psychological distress, as well as mood disorders among college students (13, 14).

Several previous studies have adopted a multi-behavior approach to examine associations with mental health outcomes. For instance, Wijbenga et al. (15) investigated the longitudinal associations of multiple health risk behaviors (including smoking, alcohol use, diet, and physical activity) with mental health in a Dutch cohort, highlighting the cumulative effects of risk behaviors. Similarly, Conry et al. (16) examined the clustering of health behaviors such as smoking, alcohol consumption, and physical inactivity in relation to psychological distress among Irish adults. In the Chinese context, studies have primarily focused on individual behaviors such as physical activity or sleep quality in relation to mental health, while the simultaneous examination of multiple behaviors—particularly including screen time, sedentary behavior, and substance use—remains limited.

Examining multiple health behaviors simultaneously, rather than in isolation, offers several important insights that cannot be obtained from single-behavior analyses. First, health behaviors often co-occur in real life; for example, individuals with higher screen time may be more likely to experience poor sleep quality and engage in unhealthy dietary behaviors (17, 18). Analyzing behaviors separately may overlook these natural clustering patterns and their cumulative or interactive effects on mental health. Second, simultaneous adjustment allows for the estimation of independent associations of each behavior while controlling for the potential confounding effects of other behaviors, thereby reducing the risk of spurious findings. Third, understanding how multiple behaviors collectively relate to mental health provides a more realistic basis for developing integrated intervention strategies, as public health programs targeting multiple risk behaviors simultaneously may be more efficient and effective than those addressing single behaviors in isolation. Fourth, this approach enables researchers to identify which behaviors remain significant when considered together, helping to prioritize targets for intervention. By adopting a multi-behavior framework, the present study aims to provide a more comprehensive understanding of the behavioral determinants of mental health among Chinese college students.

Despite the valuable contributions of these existing studies, several gaps remain. First, most multi-behavior studies have been conducted in Western populations, and findings may not be directly generalizable to Chinese college students given the distinct sociocultural context, lifestyle patterns, and mental health landscapes. Second, while previous research often focused on traditional risk behaviors such as smoking and drinking, emerging behaviors such as prolonged screen time and sedentary behavior—which are particularly relevant to the current generation of college students—have received less attention in multi-behavior frameworks. Third, few studies have simultaneously incorporated both positive wellbeing indicators (e.g., personal wellbeing) and negative mental health outcomes (e.g., depression, anxiety, stress) within the same analytical model, limiting the ability to understand the comprehensive associations across the full spectrum of mental health.

Consequently, this study aims to systematically analyze the combined characteristics of health-protective and health-risk behaviors among college students from an integrated behavioral-pattern perspective, as well as their multidimensional associations with mental health. This study is innovative in that (1) it investigates a comprehensive set of health behaviors—including physical activity, screen time, sedentary behavior, sleep quality, smoking, and alcohol use—within a single analytical framework among Chinese college students; (2) it simultaneously examines associations with both positive (personal wellbeing) and negative (depression, anxiety, stress) mental health outcomes; and (3) it provides empirical evidence from a Chinese sociocultural context, which has been underrepresented in the multi-behavior literature. By doing so, this study aims to offer a more holistic understanding of the relationship between multiple health behaviors and mental health, and to inform culturally tailored interventions for college student populations in China.

## 2 Method

### 2.1 Study design and participants

This study employed a cross-sectional survey design to investigate the relationship between health behaviors and mental health among college students. A stratified random sampling method was used to select fourteen universities across fourteen cities in Shandong and Guangdong provinces. Within each university, stratification was performed based on academic year (freshman through senior and postgraduate levels) and major category (physical education, health-related disciplines, and others). The inclusion criteria for participants were: (1) Currently enrolled as a college student (age ≥ 18 years); (2) Free from motor impairments; (3) Capable of understanding and completing the questionnaire in Chinese; and (4) Able to access and complete the electronic questionnaire via mobile device or computer. Exclusion criteria included: (1) Individuals with severe cognitive impairments or emotional/psychological disorders; (2) Those with mobility limitations or physical disabilities; and (3) individuals who did not provide informed consent.

The sample size was estimated using G^*^Power 3.1 software. Based on an effect size (*f*^2^ = 0.08) derived from previous similar studies (15), with the statistical power set at 0.80 and α at 0.05, a minimum of 196 participants was deemed necessary to achieve adequate statistical power. To account for potential nonresponse and invalid questionnaires during the actual survey process, an estimated attrition rate of 30% was considered, leading to a targeted a minimum sample size of 280 participants to ensure the reliability of the statistical analysis. Ultimately, 543 questionnaires were distributed, resulting in 521 valid responses, which yields a valid response rate of 96.1%. A total of 22 questionnaires were excluded based on the following criteria: 12 due to incomplete responses (more than 20% missing data), 6 due to logically inconsistent or patterned responses, and 4 due to completion time less than 5 min.

### 2.2 Procedure and quality control

This study was conducted in accordance with the Declaration of Helsinki, as established by the World Medical Association (19) and received approval from the Medical Research Ethics Committee of Shenzhen University (Ref. No. PN-202400172). The questionnaire was developed with reference to relevant domestic and international studies, incorporating multiple survey instruments tailored to the research objectives. These included a demographic questionnaire, the International Physical Activity Questionnaire (IPAQ), the Sedentary Behavior Questionnaire (SBQ), the Pittsburgh Sleep Quality Index (PSQI), the Smoking Behavior Questionnaire, the Alcohol Use Questionnaire, the Personal Wellbeing Index (PWI), and the Depression Anxiety Stress Scales (DASS-21). To ensure reliability and validity, a pilot survey was conducted among 120 college students, thereby meeting the minimum sample size criterion of at least five participants per item. The pilot study confirmed adequate internal consistency and construct validity, leading to the revision and refinement of any ambiguous or unclear items.

Formal Survey. During the formal survey phase, a team of trained investigators conducted on-site survey sessions organized by class in the participating universities. To ensure sample representativeness, participants were recruited through official channels such as the university's Department of Physical Education and student unions. Investigators clearly explained the purpose, significance, and requirements of the survey to the students, ensuring thorough understanding and accurate completion. The questionnaires were distributed via a professional online survey platform, with standardized instructions provided uniformly.

Data Collection and Management. After collection, all questionnaires were individually reviewed by the research team. Invalid responses were excluded based on the following criteria: (1) incomplete questionnaires with more than 20% missing data; (2) obviously patterned or logically inconsistent responses (e.g., identical answers across all items); and (3) completion time less than 5 min, which was considered insufficient for careful reading and response. A total of 22 invalid questionnaires were excluded from the analysis. Valid data were entered and organized in Excel for preliminary data cleaning and preparation for subsequent statistical analyses. To ensure accuracy, data were independently double-entered by two members of the research team and cross-checked for consistency.

### 2.3 Measures

#### 2.3.1 Physical activity

PA was measured by using the Chinese short version of the International Physical Activity Questionnaire (20, 21). IPAQ-C consisted of 6 items, which asked participants to report their PA level with three intensities (vigorous, moderate and light). Corresponding to each intensity, participants were asked to indicate how often per week and how long each time for performing these activities in the past seven days. This questionnaire included items such as “During the last 7 days, on how many days did you engage in moderate physical activities like carrying light loads, bicycling at a regular pace, or doubles tennis? (Do not include easy walking)”, and “how much time did you usually spend doing moderate physical activities on one of those days?” Based on the reported frequency and duration, the total weekly physical activity volume for each participant was calculated by summing the weighted duration across all three intensity levels.

#### 2.3.2 Sleep quality

Sleep quality was assessed using the Pittsburgh Sleep Quality Index (PSQI) (22, 23), which has been validated in Chinese populations and demonstrates good reliability and validity (24). As a standardized instrument widely used in sleep research, the PSQI comprises 19 self-rated items and 5 other-rated items, with 18 self-rated items contributing to the total score. These items are grouped into seven components: subjective sleep quality, sleep latency, sleep duration, sleep efficiency, sleep disturbances, use of sleep medication, and daytime dysfunction. Each component is scored on a scale from 0 to 3, and a global score (ranging from 0 to 21) is derived by summing the component scores. According to conventional cutoffs, a total score > 7 indicates poor sleep quality, a score ≤ 4 reflects good sleep quality, and scores between 5 and 7 suggest moderate sleep quality, with higher scores representing poorer sleep quality. In addition to the global score, this study also examined self-reported average continuous sleep duration per night (hours/night) as an important supplementary indicator of sleep patterns.

#### 2.3.3 Duration of sedentary behavior

SB among college students was assessed using the Sedentary Behavior Questionnaire (SBQ) (25). Grounded in the concept of metabolic equivalents (METs), the questionnaire defines SB as routine activities with an energy expenditure of less than 1.5 METs, encompassing typical low-energy behaviors such as watching television or using electronic devices while seated. The instrument employs a context-specific design to collect data on sedentary time (hours per day) across two typical contexts: weekdays and weekend days. Participants were asked to self-report their average daily sedentary time in these two contexts based on their recent lifestyle patterns. The mean daily sedentary time for the previous week (hours per day) was calculated using a weighted formula: (weekday sedentary time × 5 + weekend day sedentary time × 2) / 7. This measurement approach facilitates the analysis of college students' SB patterns from a temporal distribution perspective, providing a quantitative basis for exploring its potential association with physical and mental health (25).

#### 2.3.4 Smoking and drinking behaviors

Smoking and alcohol use were assessed via self- report using a binary coding approach (yes/no). Participants were classified as “smokers” if they reported any current smoking (including daily or occasional smoking), and as “non-smokers” if they reported no smoking at all. Similarly, participants were classified as “alcohol users” if they reported any current alcohol consumption (including drinking on special occasions or regularly), and as “non-users” if they reported no alcohol consumption. This simplified strategy enhances response compliance and operational efficiency while reducing participant burden, and is consistent with established methodologies commonly used in prior research (26, 27).

#### 2.3.5 Wellbeing

The Personal Wellbeing Index (PWI) was employed to assess college students' subjective wellbeing levels. Originally developed by Campbell et al. in 1976 (28), the PWI scale systematically evaluates individuals' satisfaction across multiple life domains (29, 30). It comprises seven items covering key domains closely related to wellbeing, including health status, emotional experiences, and social relationships. Using a five-point Likert scale, respondents indicate their level of agreement ranging from “very dissatisfied” (1 point) to “very satisfied” (5 points). The total score is calculated by summing all item scores, with higher scores indicating stronger subjective wellbeing. The scale has demonstrated good cross-cultural applicability and possesses ideal criterion and construct validity. In the context of this study, the PWI exhibited satisfactory psychometric properties (Cronbach's α = 0.89, CFI = 0.943, TLI = 0.941, RMSEA = 0.052), confirming its reliability and validity in consistently and effectively measuring individuals' subjective wellbeing.

#### 2.3.6 Mental and emotional state

The mental and emotional state was assessed using the 21-item Depression Anxiety Stress Scales (DASS-21) (31–33). The scale consists of three subscales with seven items each, measuring symptoms of depression, anxiety, and stress on a four-point Likert scale ranging from “Did not apply to me at all” to “Applied to me very much or most of the time.” In the current study, the DASS-21 demonstrated satisfactory psychometric properties: internal consistency was 0.83 for depression, 0.89 for anxiety, and 0.91 for stress, with an overall Cronbach's α of 0.90. Confirmatory factor analysis indicated acceptable model fit (CFI = 0.932–0.944, TLI = 0.922–0.948, RMSEA = 0.041–0.056), supporting its construct validity for assessing mental health in this sample.

It is important to note that the Personal Wellbeing Index (PWI) and the Depression Anxiety Stress Scales (DASS-21) capture conceptually distinct aspects of mental health. The PWI assesses subjective wellbeing, reflecting individuals' overall satisfaction across multiple life domains (e.g., health, relationships, and future security), and is generally considered a positive indicator of mental health. In contrast, the DASS-21 measures negative emotional states, including depression, anxiety, and stress. Although these constructs are related, they represent different dimensions of mental health: wellbeing reflects a broader and more stable evaluation of life satisfaction, whereas depression, anxiety, and stress capture more transient negative affective experiences.

#### 2.3.7 Demographic covariates

Relevant covariate data were collected using a self-administered demographic questionnaire, which covered fundamental sociodemographic characteristics including gender, academic year, major, family residence location, and family economic status. These variables were subsequently incorporated as covariates in statistical models based on prior literature demonstrating their potential associations with health behaviors and mental health outcomes among college students (34–36).

### 2.4 Statistical analyses

Data were processed and analyzed using SPSS 27.0. The procedures included the following steps: First, data quality control was performed, including examining distributions, identifying outliers, and handling missing values. Descriptive statistics were then used to summarize sample characteristics, with continuous variables reported as mean ± standard deviation and categorical variables as frequencies (percentages). Comparative analyses were conducted to examine group differences in key variables across demographic characteristics (e.g., gender, academic year, major). Subsequently, multiple regression analyses were performed to assess the associations between health behaviors and physical and mental health outcomes, including wellbeing index, depression, anxiety, and stress scores. Covariates such as age, gender, academic year, major, economic status, and self-rated health were controlled for in the models. All *a priori* selected covariates (age, gender, academic year, major, family income level, BMI) were retained in the final models based on theoretical considerations, regardless of statistical significance, to control for potential confounding effects. Multicollinearity among independent variables was assessed using variance inflation factor (VIF), with VIF values below 5 indicating no significant multicollinearity concerns. All analyses used two-tailed tests, with statistical significance set at *P* < 0.05.

## 3 Results

### 3.1 Sample characteristics

The study included 521 college students (55.7% male) with a mean age 21.33 ± 1.21 years. Baseline characteristics are summarized in Table 1. The sample was predominately composed of sports majors (46.4%) and seniors (41.8%), with most participants reporting a medium level of economic status (60.8%). Key health behavior indicators showed that 52.6% of participants consumed alcohol and 45.9% were current smokers. The average sleep duration was 7.86 ± 2.08 h per night, with a daily sedentary time of 8.88 ± 6.37 h. PA levels averaged 240.18 ± 307.81 min per week across all intensities. Mental health measures indicated a mean wellbeing index score of 3.71 ± 0.81. DASS-21 subscale scores were 6.61 ± 6.41 for depression, 6.86 ± 6.20 for anxiety, and 7.22 ± 6.22 for stress. Self-rated health averaged 3.69 ± 0.887 on a 5-point scale.

**Table 1:** Characteristics of the study sample (*n* = 521).

Variables	Mean (SD) or Frequency (percentage)
Age (years)	21.33 (1.2)
Gender	
Male	290 (55.7)
Female	231 (44.3)
Body mass index (kg/m^2^)	20.29 (3.4)
Weight status	
Non-overweight	461 (88.5)
Overweight	60 (11.5)
Academic majors	1.86 (0.9)
Sports-related	242 (46.4)
Health-related	110 (21.1)
Others	169 (32.4)
Academic year	
Freshman	38 (7.3)
Sophomore	89 (17.1)
Junior	135 (25.9)
Senior	218 (41.8)
Postgraduate	41 (7.9)
Family socioeconomic status	
Low	141 (27.1)
Medium	317 (60.8)
High	63 (12.1)
Self-rated health score (1–5)	3.69 (0.9)
Physical activity (min/week)	278.16 (36.9)
Sleep duration (hours/day)	7.84 (2.1)
Screen time (hours/day)	5.69 (2.4)
Alcohol consumption	
Yes	274 (52.6)
No	247 (47.4)
Smoking status	
Yes	239 (45.9)
No	282 (54.1)
Personal wellbeing index score (1–5)	3.71 (0.8)
Depression score (0–21)	6.61 (6.4)
Anxiety score (0–21)	6.86 (6.2)
Stress score (0–21)	7.22 (6.2)

### 3.2 Gender differences in the health behaviors and mental health of the participants

Table 2 presents the results of gender differences in health behaviors and mental health among college students. Independent samples *t*-tests indicated no statistically significant gender differences in personal wellbeing, stress, depression, anxiety, sleep quality (SQ), screen time, sleep duration, or PA (all *P* > 0.05).

**Table 2:** Results of gender differences in the health behaviors and mental health of the participants.

Variables	Gender	Mean (SD)	*t* value	95%*CI*	*P* value
Personal wellbeing index	Male (*n* = 290)	3.79 (0.82)	*t*_519_ = 2.52	0.039–0.319	0.12
	Female (*n* = 231)	3.60 (0.79)			
Depression	Male (*n* = 290)	7.08 (6.71)	*t*_519_ = 1.88	−0.46–2.17	0.06
	Female (*n* = 231)	6.02 (5.98)			
Anxiety	Male (*n* = 290)	7.24 (6.46)	*t*_519_ = 1.58	−0.20–1.91	0.11
	Female (*n* = 231)	6.38 (5.80)			
Stress	Male (*n* = 290)	7.40 (6.57)	*t*_519_ = 0.74	−0.67–1.49	0.46
	Female (*n* = 231)	6.99 (5.77)			
Sleep quality	Male (*n* = 290)	6.47 (2.07)	*t*_519_ = −1.37	−0.62–0.11	0.17
	Female (*n* = 231)	6.72 (2.12)			
Sleep duration	Male(*n* = 290)	7.91 (1.91)	*t*_519_ = 0.89	−0.19–0.53	0.37
	Female(*n* = 231)	7.75 (2.22)			
Screen time	Male (*n* = 290)	5.64 (2.48)	*t*_519_ = −0.57	−0.53–0.29	0.57
	Female (*n* = 231)	5.76 (2.28)			
Physical activity	Male (*n* = 290)	281.23 (28.29)	*t*_519_ = 0.23	−51.85–65.80	0.82
	Female (*n* = 231)	274.25 (48.12)			

### 3.3 Correlation analysis of multiple health behaviors and mental health

After adjusting for covariates, regression analyses revealed specific associations between health behaviors and psychological outcomes (see Table 3): both alcohol consumption *(*β = −0.16, *P* = 0.040) and screen time (β = −0.03, *P* = 0.036) were negatively correlated with personal wellbeing; smoking showed negative associations with stress (β = −1.37, *P* = 0.020) and depression (β = −1.44, *P* = 0.016), whereas screen time (β_stress_ =0.24, *P*_stress_ = 0.030; β_depression_ =0.32, *P*_depression_ = 0.005) was positively correlated with these two outcomes; for anxiety, only screen time (β =0.25, *P* = 0.020) demonstrated a significant positive association. Notably, although the magnitude of the association between screen time and personal wellbeing was modest, the consistent associations observed across multiple mental health outcomes—including stress, depression, and anxiety—may suggest a potentially broader relationship between screen time and mental health. The regression models explained 12.4% (Personal Wellbeing), 13.2% (Stress), 13.8% (Depression), and 12.6% (Anxiety) of the variance in mental health outcomes, as indicated by adjusted R^2^ values. Overall, the models accounted for approximately 12–14% of the variance. This level of explained variance is consistent with previous cross-sectional studies examining mental health among university students, where relatively modest adjusted R^2^ values are typically observed, often ranging from low to moderate levels (e.g., approximately 5%−18%) (37–39). Considering the complex and multifactorial nature of mental health (40), these model fit statistics can be regarded as acceptable.

**Table 3:** Results of the relationship between various healthy behaviors of college students and mental health.

Variables	Wellbeing		Stress		Depression		Anxiety	
	*β (95%CI)*	*P*	*β (95%CI)*	*P*	*β (95%CI)*	*P*	*β (95%CI)*	*P*
Intercept	1.10 (-4.43–6.62)	0.700	33.62 (-8.28–75.53)	0.120	21.64 (-21.25–64.53)	0.320	29.01 (-12.47–70.48)	0.170
Concomitant variable								
Age (year)	0.12 (-0.17–0.41)	0.420	−1.13 (-3.33–1.07)	0.310	−0.48 (-2.73–1.78)	0.680	−0.90 (-3.08–1.28)	0.420
Gender (Ref.: male)								
Female	−0.04 (-0.20–0.12)	0.610	−1.72 (-2.93–−0.51)	0.006	−2.41 (-3.64–−1.17)	< 0.001	−2.08 (-3.28–−0.88)	0.001
Grade (Ref.: freshman)								
Sophomore	−0.03 (-0.45–0.39)	0.880	1.52 (-1.65–4.70)	0.350	1.41 (-1.84–4.66)	0.390	1.39 (-1.75–4.53)	0.380
Junior	−0.22 (-0.87–0.43)	0.510	2.64 (-2.27–7.55)	0.290	1.35 (-3.67–6.38)	0.590	2.18 (-2.68–7.04)	0.380
Senior	−0.38 (-1.29–0.53)	0.410	3.89 (-3.01–10.80)	0.270	2.28 (-4.79–9.35)	0.530	3.19 (-3.65–10.02)	0.360
Graduate student	−0.39 (-1.85–1.07)	0.600	4.55 (-6.51–15.61)	0.420	1.77 (-9.55–13.09)	0.760	3.05 (-7.89–14.0)	0.580
Major (Ref.: P.E.)								
Health Professionals	−0.23 (-0.42–−0.04)	0.020	3.64 (2.20–5.08)	< 0.001	3.41 (1.94–4.89)	< 0.001	3.61 (2.18–5.03)	< 0.001
Others	−0.21 (-0.38–−0.04)	0.013	0.21 (-1.07–1.49)	0.750	0.02 (-1.29–1.33)	0.970	0.04 (-1.23–1.31)	0.950
Family income level (Ref.: low)								
Middle	0.23 (0.07–0.39)	0.004	0.28 (-0.93–1.50)	0.650	0.02 (-1.23–1.26)	0.980	0.17 (-1.03–1.38)	0.770
High	0.57 (0.33–0.81)	< 0.001	−0.42 (-2.21–1.38)	0.650	−0.24 (-2.08–1.59)	0.790	−0.24 (-2.01–1.54)	0.790
BMI (kg/m^2^)	0.03 (0.01–0.05)	0.007	−0.33 (-0.49–−0.16)	< 0.001	−0.38 (-0.55–−0.21)	< 0.001	−0.34 (-0.51–−0.18)	< 0.001
Health behavior								
Smoking (Ref.: no)	0.10 (-0.05–0.25)	0.180	-**1.37 (-2.51 – -0.23)**	**0.020**	-**1.44 (-2.61 – -0.27)**	**0.016**	−0.93 (-2.07–0.2)	0.110
Alcohol use (Ref.: no)	-**0.16 (-0.31 – -0.01)**	**0.040**	0.36 (-0.77–1.49)	0.530	0.33 (-0.83–1.48)	0.580	0.01 (-1.11–1.13)	0.980
Sleep duration	−0.02 (-0.05–0.02)	0.350	0.04 (-0.22–0.30)	0.750	0.05 (-0.22–0.32)	0.073	0.11 (-0.15–0.37)	0.400
Screen time	-**0.03 (-0.06 – -0.002)**	**0.036**	**0.24 (0.02–0.46)**	**0.030**	**0.32 (0.10–0.54)**	**0.005**	**0.25 (0.04–0.47)**	**0.020**
Physical activity	0.01 (-0.01–0.01)	0.950	0.01 (-0.002–0.001)	0.750	0.01 (-0.002–0.001)	0.630	−0.001 (-0.002–0.001)	0.490

## 4 Discussion

This study investigated the status and interrelationships among multiple health behaviors and psychological wellbeing among college students in Shandong and Guangdong provinces. Overall, the findings suggest a coexistence of adequate sleep duration, suboptimal sleep quality, and prevalent unhealthy lifestyle behaviors, alongside varying levels of psychological distress.

With regard to sleep, the mean sleep quality score was 6.58 ± 2.09. According to conventional cutoffs (24) ( ≤ 4 indicating good sleep quality, 5–7 moderate, and >7 poor), this value falls within the moderate range, suggesting suboptimal sleep quality on average. Actual sleep duration averaged 7.84 ± 2.06 h per night, which falls within the recommended range of 7–9 h for young adults (41). These findings indicate that although sleep duration appears adequate, sleep quality remains an area requiring improvement.

In terms of health-related behaviors, screen time averaged 5.69 ± 2.39 h per day, exceeding levels generally recommended for recreational use in health promotion contexts. Although no standardized adult threshold has been established, accumulating evidence suggests that higher screen exposure is associated with adverse physical and mental health outcomes (42). In addition, alcohol consumption and smoking were relatively prevalent among participants, with 52.6% and 45.9% reporting these behaviors, respectively. These findings are consistent with previous studies indicating widespread unhealthy lifestyle behaviors among college students (43), potentially influenced by academic stress, social adaptation challenges, and modern lifestyle patterns.

Regarding mental health status, the mean personal wellbeing index indicated a relatively satisfactory level. However, scores for depression, anxiety, and stress suggested mild to moderate psychological distress, consistent with prior research highlighting increasing mental health challenges among university students (44).

In terms of associations between sleep and mental health, no significant relationships were observed between sleep duration and any mental health outcomes, including wellbeing, stress, depression, and anxiety. This may reflect the complexity of sleep–mental health relationships, which are influenced by multiple dimensions such as sleep quality, circadian rhythms, and individual variability (26). It also suggests that sleep duration alone may be insufficient to capture the full impact of sleep on psychological functioning.

For alcohol and smoking behaviors, alcohol consumption was negatively associated with personal wellbeing but not significantly related to stress, depression, or anxiety. This may indicate that alcohol use is more closely linked to global life satisfaction rather than short-term emotional states. Smoking showed negative associations with stress and depression, which may reflect maladaptive coping strategies such as self-medication of negative emotions (45). However, smoking was not significantly associated with overall wellbeing, suggesting limited influence on global life satisfaction.

Screen time showed a more complex pattern of associations with mental health outcomes, being related to both positive (wellbeing) and negative (stress, depression, and anxiety) indicators. This pattern is consistent with behavioral activation theory (46, 47), which suggests that reduced engagement in health-promoting activities may contribute to poorer mental health. Prolonged screen use may displace physical activity and face-to-face social interaction and may also disrupt circadian rhythms, thereby influencing both positive and negative psychological states (48, 49). From a public health perspective, reducing excessive screen exposure may represent a meaningful target for mental health interventions among college students.

Although the association between screen time and personal wellbeing was statistically significant, the effect size was small (β = −0.03), which is not unexpected given the multifactorial nature of wellbeing. In contrast, stronger associations were observed for stress, depression, and anxiety (β ranging from 0.24 to 0.32). From a population health perspective, even small effects may be meaningful, as modest behavioral changes at the individual level may translate into substantial benefits at the population level (50). These findings suggest that the observed associations may have both clinical and practical relevance.

The relatively small effect size for wellbeing may also reflect differences in measurement sensitivity. The Personal Wellbeing Index (PWI), which captures global life satisfaction across multiple domains, may be less sensitive to screen exposure compared to negative affective states measured by the DASS-21. This may partly explain the observed variation in effect sizes across outcomes.

Despite these insights, several limitations should be acknowledged. The cross-sectional design limits causal inference. Self-reported measures may introduce recall and social desirability bias. The sample was restricted to two provinces in China, limiting generalizability. In addition, the regression-based approach does not account for potential interactions or synergistic effects among health behaviors. Future studies should consider longitudinal designs, objective measurements, and advanced analytical approaches such as interaction modeling or latent class analysis to better capture behavioral patterns and their combined effects on mental health. Finally, the PWI and DASS-21 reflect distinct dimensions of mental health—positive life evaluation versus negative affect—which may partly explain the differential associations observed. Future research should incorporate broader mental health indicators to further clarify these pathways.

## 5 Conclusion

This study investigated the associations between multiple health behaviors and mental health outcomes among Chinese college students. The findings revealed that screen time was consistently associated with poorer mental health, showing negative associations with personal wellbeing and positive associations with stress, depression, and anxiety. Alcohol consumption was negatively associated with personal wellbeing but showed no significant associations with stress, depression, or anxiety. Smoking was negatively associated with stress and depression, suggesting it may serve as a maladaptive coping mechanism, though it was not associated with personal wellbeing. In contrast, physical activity and sleep duration did not show statistically significant associations with mental health outcomes in the fully adjusted models.

Based on these findings, targeted and practical interventions are warranted. Universities should prioritize strategies to reduce excessive screen time, such as promoting digital wellbeing education and encouraging structured offline activities. In addition, preventive efforts should address alcohol consumption and smoking by strengthening health education, implementing campus-based restrictions, and providing healthier coping alternatives, such as stress management programs and psychological counseling services.

Importantly, given that physical activity and sleep duration were not significantly associated with mental health outcomes in the fully adjusted models, these findings should be interpreted with caution. The absence of significant associations may reflect limitations in measurement (e.g., lack of information on intensity, quality, or behavioral patterns) or residual confounding. Therefore, future research should employ more comprehensive and objective assessments to clarify these relationships. Nevertheless, maintaining adequate physical activity and sleep remains essential for overall health.

At the family and societal levels, coordinated efforts are needed to reinforce positive health behaviors, improve access to mental health resources, and enhance health literacy. Such multi-level approaches may contribute to more effective promotion of mental health among college students.

## Data Availability

The original contributions presented in the study are included in the article/supplementary material, further inquiries can be directed to the corresponding authors.
